# Arritmias Cardíacas e COVID-19: Lado a Lado na Pandemia

**DOI:** 10.36660/abc.20210810

**Published:** 2021-11-01

**Affiliations:** 1 Universidade de São Paulo Faculdade de Medicina Hospital das Clínicas São Paulo SP Brasil Instituto do Coração do Hospital das Clínicas da Faculdade de Medicina da Universidade de São Paulo, São Paulo, SP – Brasil

**Keywords:** Arritmias Cardíacas, COVID-19, Pandemias

A atual pandemia causada pelo novo coronavírus (SARS-COV-2), detectado inicialmente em dezembro de 2019 na cidade de Wuhan, China, mudou a dinâmica da saúde em todo o mundo. De acordo com os últimos registros da Organização Mundial da Saúde, o número de casos de COVID-19 no mundo passa de 224 milhões, com o total de mortos superando 4,6 milhões.^1^ O aumento exponencial no número de casos gerou alta demanda nos serviços de saúde, e novos dados sobre a doença e seu tratamento são constantemente atualizados.

O acometimento mais marcante na COVID-19 faz-se sobre o sistema respiratório. Entretanto, outros sistemas são frequentemente acometidos, como o sistema cardiovascular, levando a injúria miocárdica e arritmias.^2,3^

A primeira descrição dos 138 casos na China mostrou que as arritmias cardíacas foram uma complicação em 16,7% do total de casos, subindo para 44,4% nos pacientes internados em unidade de terapia intensiva (UTI), sem distinção quanto ao tipo das arritmias encontradas.^3^ Em outro relato italiano de fevereiro de 2020, observou-se um aumento no número de paradas cardíacas extra-hospitalares de 58% em relação ao mesmo período do ano anterior. Porém, o estudo não apresentou registros dos ritmos cardíacos no atendimento desses pacientes, além da não confirmação de infecção pelo SARS-COV-2.^4^ Nesse contexto, novos estudos foram publicados com o objetivo de melhor compreender a associação entre COVID-19 e arritmias.

Os mecanismos fisiopatológicos para as arritmias permanecem incertos na infecção pelo novo coronavírus. As hipóteses são de internalização e redução de receptores da enzima conversora de angiotensina 2, hiperativação inflamatória e imune com aumento de citocinas, disfunção endotelial, hipoxemia, hiperativação simpática ou disautonomia, além de distúrbios hidroeletrolíticos, os quais, em conjunto, causariam alterações na despolarização e repolarização dos miócitos, principalmente nos casos mais graves da doença.^5,6^ A presença de comorbidades prévias e eventual substrato arritmogênico associado, além do uso frequente de medicações que aumentam o intervalo QT podem predispor à arritmia.^2,3,7^

As arritmias apresentadas pelos pacientes são variadas, com ocorrência de bradicardias sinusais, bloqueio atrioventricular, e taquicardias (supraventricular e ventricular). Em um estudo em hospital americano incluindo 700 pacientes que testaram positivo para SARS-COV-2, com 11% de internados em UTI, verificaram-se 53 eventos de arritmias, incluindo 25 casos de fibrilação atrial, nove casos de bradicardia, 10 casos de taquicardia ventricular não sustentada, além de nove paradas cardiorrespiratórias sendo seis em ritmo de atividade elétrica sem pulso, dois em assistolia e um em torsades de pointes.^8^ Em uma outra análise de dados mundiais,^9^ englobando 4526 pacientes hospitalizados pela COVID-19 em 12 países, incluindo o Brasil e a atual publicação, 827 apresentaram arritmias durante a internação, dos quais 81,8% tiveram taquicardias supraventriculares (principalmente fibrilação atrial), 20,7% tiveram taquicardias ventriculares e 22,6% tiveram bradicardias, com uma taxa de incidência de arritmias de 12,9%. Nesse registro, a presença de arritmias esteve associada a um pior prognóstico, com uma maior taxa de morbimortalidade.^9^

No atual cenário, informações sobre registros brasileiros são relevantes. Pimentel et al.^10^ trouxeram informações de 241 pacientes internados em hospital terciário com diagnóstico de COVID-19, confirmado por PCR, baseadas em revisão de prontuário. Foram constatados idade média de 57,8 anos, necessidade de UTI em 35,3% dos casos, uso de ventilação mecânica em 58,8%, e taxa de mortalidade de 26,6%. A incidência de arritmias nessa população foi de 8,7%, sendo 76,2% taquicardias supraventriculares, 14,3% taquicardias ventriculares sustentadas e 9,5% bradicardias. Houve oito casos de parada cadiorrespiratória em UTI, com apenas dois em ritmo chocável (fibrilação ventricular / taquicardia ventricular sem pulso). Dentre as comorbidades elencadas, apenas a insuficiência cardíaca prévia mostrou-se como fator de risco significativo para arritmias. Pacientes com arritmias durante a internação tiveram uma maior chance de evolução a óbito (*hazard ratio*, 3,4, IC 95% 1,8-6,7, p <0,05), em concordância com achados internacionais.^9^

Dessa forma, o presente estudo agrega dados sobre a associação entre infecção pelo SARS-COV-2 e as arritmias cardíacas, apesar de um número limitado de pacientes em registro unicêntrico. Ademais, vale destacar que a maioria dos estudos realizados mundialmente até o momento consiste em revisão de prontuário, com o diagnóstico das arritmias por meio de monitores, nem sempre documentadas com ECG de 12 derivações, além da baixa taxa de utilização de telemetria em leitos de enfermaria, podendo subestimar sua real incidência.^8–10^ Estudos multicêntricos com maior número de pacientes de diversos níveis de gravidade são necessários para uma melhor compreensão da relação causa-consequência entre arritmias e mortalidade, e definição das medidas de prevenção, vigilância e tratamento.
