# Supplementary material for: Arritmias Cardíacas e COVID-19: Lado a Lado na Pandemia
Source: Arq Bras Cardiol. 2021 Nov 1;117(5):1016–7. [Article in Portuguese] doi: 10.36660/abc.20210810 (PMC8682085; doi:10.36660/abc.20210810)
Supplement: Translation

# Translation: Cardiac Arrhythmias and COVID-19: Side-By-Side in the Pandemic

**Affiliations:** 1 São Paulo SP Brazil Instituto do Coração do Hospital das Clínicas da Faculdade de Medicina da Universidade de São Paulo, São Paulo, SP – Brazil

**Keywords:** Arrhythmias, Cardiac, COVID-19, Pandemics

The current pandemic caused by the new coronavirus (SARS-CoV-2), which was first detected in Wuhan, China, in December 2019, has changed healthcare services worldwide. According to recent reports of the World Health Organization, there are more than 224 million cases of COVID-19 in the world, with deaths surpassing 4.6 million.^1^ The exponential increase in the number of cases has caused an impact on healthcare demand, and data on COVID-19 and its treatment have been constantly updated.

Although the respiratory system is the most affected by the SARS-CoV-2, other systems are commonly involved, including the cardiovascular system, leading to myocardial injury and arrythmias.^2,3^

In the first report of the 138 cases in China, cardiac arrhythmias were observed in 16.7% of total cases, and in 44.4% of intensive care unit (ICU) patients, with no distinction in the type of arrhythmias.^3^ In another report from Italy, published in February 2020, the number of cases of out-of-hospital cardiac arrest increased by 58% as compared with the number of cases identified during the same period in the previous year. However, the study neither presented detailed information on heart rhythm nor confirmed SARS-CoV-2 infection. In this context, recent studies have been conducted aiming at better understanding the association between COVID-19 and cardiac arrhythmias.

The pathophysiological mechanisms of arrhythmia are still uncertain in COVID-19; hypotheses include internalization and reduction of angiotensin-converting enzyme 2 receptors, inflammatory and immune hyperactivation with increased cytokines, endothelial dysfunction, hypoxemia, sympathetic hyperactivation or dysautonomia, which altogether, cause changes in myocyte depolarization and repolarization, mainly in severe cases of the disease.^5,6^ The presence of previous comorbidities and eventual arrhythmogenic substrate, combined with the frequent use of drugs that prolong the QT interval, may predispose to arrhythmia.^2,3,7^

Patients may present many types of arrhythmias, with occurrence of sinus bradycardia, atrioventricular block, and tachycardias (ventricular and supraventricular). In a study conducted in an American hospital with 700 patients with positive result for SARS-CoV-2, 11% received ICU care, there were 53 arrhythmia events, including 25 cases of atrial fibrillation, nine cases of bradycardia, 10 cases of nonsustained ventricular tachycardia, and nine cases of cardiorespiratory arrest, six of them pulseless electrical activity, two asystolic cardiac arrest and one case of torsades de pointes.^8^ In another analysis of world data,^9^ encompassing 4,526 patients hospitalized for COVID-19 in 12 countries, including Brazil and the current publication, 827 patients had arrhythmias during hospitalization; 81.8% of them had supraventricular tachycardia (mainly atrial fibrillation), 20.7% had ventricular tachycardia, and 22.6% had bradycardia, with an incidence of arrhythmia of 12.9%. In this study, the presence of arrhythmias was associated with a poor prognosis, with higher rate of morbidity and mortality.^9^

In the current scenario, data on Brazilian registries are relevant. Pimentel et al.^10^ presented data of 241 patients admitted to a tertiary hospital with a diagnosis of COVID-19, confirmed by real-time PCR, based on medical record review. Mean age of participants was 57.8 years, 35.5% of the cases required intensive care therapy, 58.8% required mechanical ventilation, and mortality rate was 26,6%. The incidence of arrhythmias was 8,7%, of which 76,2% were supraventricular tachycardia, 14,3% were sustained ventricular tachycardia and 9,5% were bradycardia. There were eight cases of cardiorespiratory arrest in the ICU, only two with a shockable rhythm (ventricular fibrillation / pulseless ventricular tachycardia). Among the comorbidities analyzed, only previous heart failure was shown to be a significant risk factor for arrhythmia. Patients who had arrhythmias during hospitalization had greater odds of death (hazard ratio, 3.4. 95%CI 1.8-6.7; p<0.05), in accordance with results of studies conducted in other countries.^9^

Thus, despite its limited number of patients and single-center registry, the present study provides data on the association between SARS-CoV-2 infection and cardiac arrhythmias. Also, it is worth pointing out that most of the studies carried out in the world consist of medical chart reviews, in which diagnosis of arrhythmias was based on rhythm monitoring, sometimes without a 12-lead electrocardiogram, and a minor use of telemetry in the ward, which may underestimate the real incidence of arrhythmias. Multicentric studies with larger number of patients including different disease severity are needed for a better association between arrhythmias and mortality, so as to define measurements of prevention, surveillance and treatment.
